# Focal Area of Low T2 Signal on MRI Scans in a Heterogeneous Uterine Leiomyoma Does Not Exclude the Possibility of Malignancy: A Report of Two Cases

**DOI:** 10.1155/crra/5388015

**Published:** 2025-04-25

**Authors:** Siegfried Hélage, Claudia Laponche, Margaux Homps, Jean-Noël Buy, Pierre-Alexandre Just, Denis Jacob, Michel Ghossain, Élisabeth Dion

**Affiliations:** ^1^Department of Radiology, Hôtel-Dieu de Paris (AP-HP), Paris, France; ^2^Department of Pathology, Hôpital Cochin (AP-HP), Paris, France; ^3^Department of Gynecological Surgery, Clinique Bizet, Paris, France; ^4^Department of Radiology, CHU Hôtel-Dieu de France, Beirut, Lebanon

**Keywords:** ADC, carcinosarcoma, diffusion-weighted imaging, endometrial stromal sarcoma, leiomyosarcoma, MMMT, MRI, smooth muscle tumor of the uterus, STUMP, uterine fibroid, uterine leiomyoma, uterine sarcoma

## Abstract

**Background:** Uterine sarcomas are uncommon malignant tumors with a grim prognosis, accounting for less than 1% of all gynecologic malignancies. Radiological series often include a limited number of patients, and diagnostic approaches can vary. While the presence of low T2 signal intensity in leiomyomas on MRI has been proposed as a criterion to exclude sarcoma, exceptions to this rule exist. We present two cases that challenge this notion.

**Case reports:** The first patient was a 48-year-old woman presenting with metrorrhagia. MRI revealed a large intramural leiomyoma characterized by extensive hypointensity on T2-weighted imaging (T2WI) and a small intraleiomyoma focus with intermediate signal intensity. Histopathological examination confirmed leiomyosarcoma. The second patient was a 51-year-old woman presenting with menometrorrhagia. MRI showed a subserosal myoma with zones of T2WI hypointensity interspersed with a region of intermediate signal intensity. Histopathological examination confirmed low-grade endometrial stromal sarcoma. In both cases, diffusion-weighted imaging (DWI) revealed an intratumoral zone of restricted diffusion, with an apparent diffusion coefficient (ADC) value ≤ 0.86 × 10^−3^ mm^2^/s.

**Conclusion:** MRI is crucial for distinguishing leiomyomas from sarcomas. We propose combining T2WI and DWI with ADC for this purpose, noting limitations in each sequence's reliability. Suggestive MRI criteria for malignancy in sarcomas are identified, emphasizing the need for comprehensive imaging analysis. In characterizing uterine smooth muscle tumors, particularly when analyzing leiomyoma variants, DWI emerges as the dominant sequence, with T2WI serving as a secondary sequence. ADC values aid in histopathological hypothesis, but caution is warranted due to overlap with benign lesions. This approach may refine preoperative diagnosis and guide therapeutic management.

## 1. Background

Uterine sarcomas, the vast majority of which are leiomyosarcomas, are exceptionally uncommon malignant tumors known for their unfavorable prognosis. They represent less than 1% of all gynecologic malignancies and approximately 3% of all malignant uterine tumors. The overall two-year survival rate is less than 50% [1, 2]. In the literature, with a few exceptions [3–5], radiological series typically include a limited number of patients. The authors of the largest series to date, comprising 156 women, concluded that MRI features potentially excluding sarcoma included the presence of an area of low T2 signal within a heterogeneous mass, regardless of DWI signal. They based their diagnostic algorithm on this “theorem,” suggesting that the diffusion sequence may be disregarded or not performed if this sign is present [4]. However, our experience, illustrated by two clinical cases, has shown that strictly adhering to this “theorem” may lead to the misclassification of true sarcomatous uterine masses, proven by histopathological examination, as simple leiomyoma variants.

## 2. Case Reports

### 2.1. Case 1

**Figure 1:**
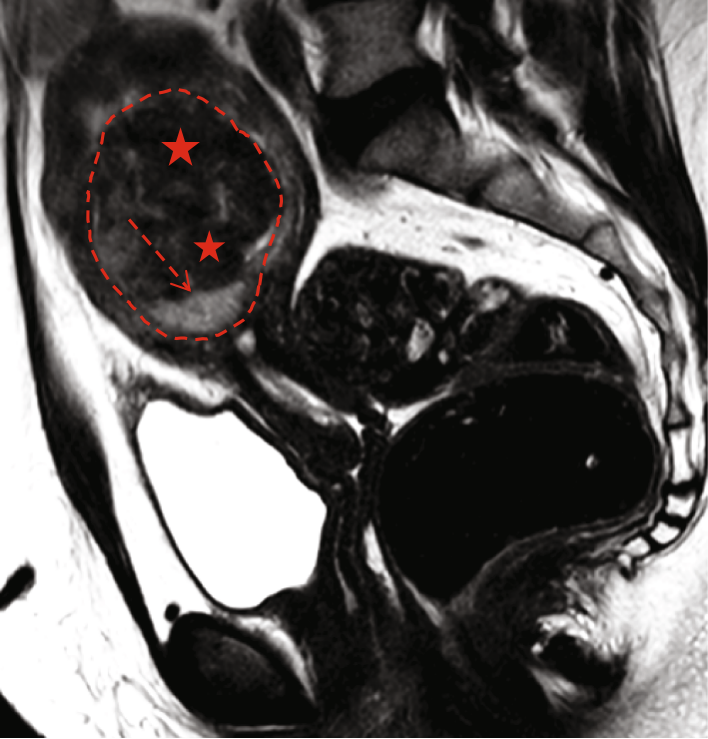
Pelvic MRI of a histopathologically diagnosed leiomyosarcoma: sagittal T2WI showing a submucosal “lookalike” leiomyoma (outlined by a red dashed line). T2WI reveals a small peripheral intraleiomyoma islet with intermediate signal intensity (dashed arrow) and a markedly predominant component with low signal (red stars) similar to that of pelvic or gluteal muscles.

**Figure 2:**
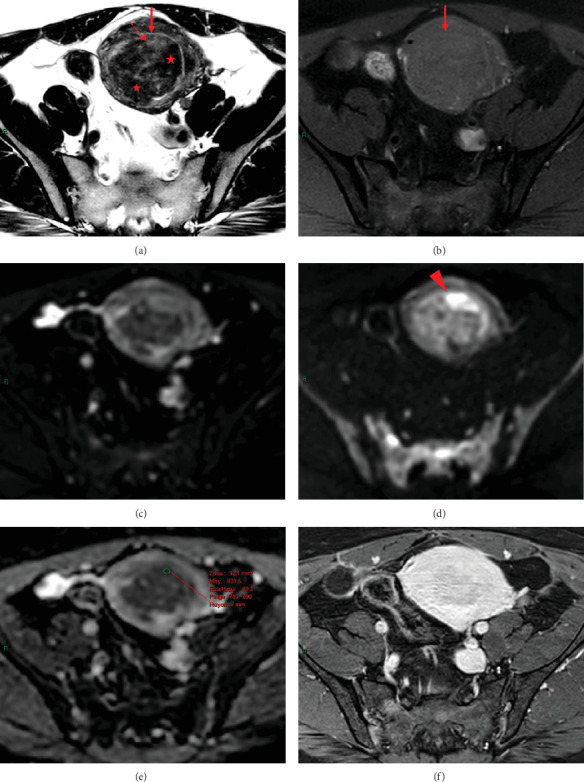
Pelvic MRI of a histopathologically diagnosed leiomyosarcoma: (a) axial T2WI, (b) axial T1WI with Fat-Sat, (c) axial b0 DWI, (d) axial b1000 DWI, (e) ADC map, and (f) axial contrast–enhanced T1WI with Fat-Sat, showing a “lookalike” leiomyoma (arrow) measuring 65 mm in greatest diameter. T2WI reveals a small peripheral intraleiomyoma islet with intermediate signal intensity (dashed arrow) and a markedly predominant component with low signal (red stars) similar to that of pelvic or gluteal muscles, without a hemorrhagic component on T1WI with Fat-Sat, which would theoretically classify the tumor as “certainly benign.” Systematic performance of DWI reveals the small intraleiomyoma islet, measuring 12 mm in greatest diameter, to exhibit high signal intensity on b1000 DWI (arrowhead) compared to the outer myometrium, with this intensity visually increasing conspicuously from the b0-weighted sequence, consistent with a low ADC value (0.832 × 10^−3^mm^2^/s).

In April 2015, a 48-year-old woman presented with metrorrhagia. A pelvic MRI performed in May 2015 revealed a large intramural uterine leiomyoma with submucosal extension, measuring 65 mm in diameter (Figure 1). The lesion was predominantly hypointense on T2WI, with a peripheral intraleiomyoma area measuring 12 mm and demonstrating intermediate signal intensity. On DWI at b1000, this intraleiomyoma area displayed marked hyperintensity with a decreased ADC value of 0.832 mm^2^/s (Figure 2). No spontaneous hyperintensity on T1WI with Fat-Sat was observed to suggest intratumoral hemorrhage. Homogeneous enhancement of the tumor was noted following contrast administration.

A multidisciplinary expert consultation led to a decision for total vaginal hysterectomy without morcellation and bilateral annexectomy, which were performed in May 2015. Histopathological examination revealed that the mass predominantly consisted of spindle cells arranged in fascicles, lacking atypical nuclei and abnormal mitosis. These surrounded a small peripheral intraleiomyoma islet containing small foci of limited coagulative tumor cell necrosis. This intraleiomyoma islet was composed of spindle cells with marked nuclear atypia and 41 mitoses per 10 high-power fields. These tumor cells were positive for smooth muscle markers, including smooth muscle actin, desmin, and h-caldesmon. Consequently, the tumor was histopathologically diagnosed as a uterine leiomyoma with an area of transformation into leiomyosarcoma, classified as FIGO Stage 1B. Adjuvant chemotherapy was not indicated. The patient is currently under regular surveillance and shows no signs of recurrence.

### 2.2. Case 2

**Figure 3:**
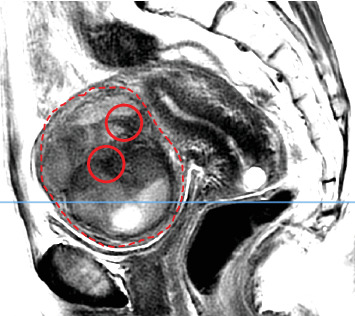
Pelvic MRI of a histopathologically diagnosed endometrial stromal sarcoma: sagittal T2WI showing an intramyometrial subserosal mass (outlined by a red dashed line). T2WI reveals an intratumoral area with intermediate signal intensity intermixed with a patchy distribution of low-signal components (red circles) similar to pelvic or gluteal muscles. The blue line represents the sectional plane of the axial view shown in Figure 4.

**Figure 4:**
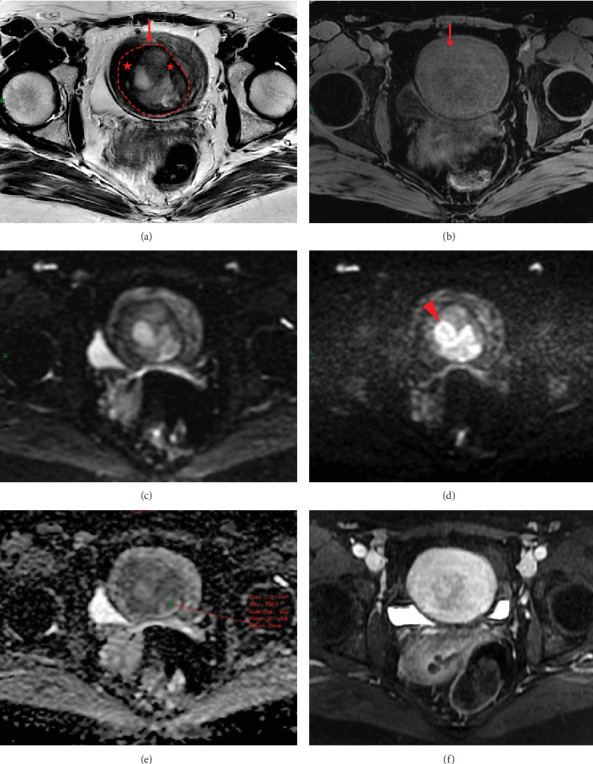
Pelvic MRI of a histopathologically diagnosed endometrial stromal sarcoma: (a) axial T2WI, (b) axial T1WI with Fat-Sat, (c) axial b0 DWI, (d) axial b1000 DWI, (e) ADC map, and (f) axial contrast–enhanced T1WI with Fat-Sat, showing a mass (arrow) measuring 80 mm in greatest diameter. T2WI reveals an intratumoral area with intermediate signal intensity intermixed with peripheral low-signal components (red stars) similar to pelvic or gluteal muscles, without a hemorrhagic component on T1WI with Fat-Sat, which would theoretically classify the tumor as “certainly benign.” Note the characteristic thin and smooth low-intensity rim (outlined by the red dashed line) on T2WI, visible around the entire mass, considered an MRI feature useful for distinguishing intramyometrial endometrial stromal sarcoma from other benign lesions [6, 7]. Systematic performance of DWI reveals this intratumoral area, measuring 32 mm in greatest diameter, to exhibit high signal intensity on b1000 DWI (arrowhead) compared to the outer myometrium, with this intensity visually increasing conspicuously from the b0-weighted sequence, consistent with a low ADC value (0.858 × 10^−3^mm^2^/s).

In January 2022, a 51-year-old woman presented with menometrorrhagia. Laboratory tests revealed an elevated plasma CA125 level. A pelvic MRI performed in February 2022 identified a subserosal corporal myoma measuring 80 mm in diameter (Figure 3). The mass exhibited zones of hypointensity on T2WI. Within the lesion, there was a 32-mm area demonstrating intermediate T2WI signal intensity, with marked hyperintensity on b1000 DWI and a reduced ADC value of 0.858 mm^2^/s (Figure 4). No spontaneous hyperintensity on T1WI with Fat-Sat was observed to suggest intratumoral hemorrhage. Following contrast administration, relatively homogeneous enhancement of the tumor was noted.

Diagnostic hysteroscopy in February 2022 revealed a slightly hypertrophied endometrium, with a hemorrhagic biopsy specimen that was histopathologically noncontributory. A multidisciplinary expert consultation led to a decision for laparotomy in April 2022, resulting in total hysterectomy and bilateral annexectomy. Histopathological examination revealed that the mass consisted of monotonous oval cells, resembling the endometrial stroma in the proliferative phase, with no cytologic atypia, mitoses, or necrosis. The tumor infiltrated the surrounding myometrium, forming a permeative tongue-like pattern of invasion without evidence of vascular invasion. Immunostaining was positive for CD10, ER, and PR. A molecular alteration identified via FISH analysis showed a rearrangement involving the *JAZF1* gene. Consequently, the tumor was histopathologically diagnosed as a low-grade endometrial stromal sarcoma, classified as FIGO Stage 1B. Adjuvant hormone therapy was not deemed necessary. The patient is currently under regular surveillance and shows no signs of recurrence.

## 3. Discussion

MRI preoperative distinction between leiomyoma and uterine sarcoma using T2WI and DWI with ADC is fundamental for therapeutic management because surgical fragmentation of sarcomas is contraindicated. Negative consequences of tumoral morcellation on the survival of patients who are found to have malignant instead of benign uterine masses are recognized, with a risk of intraperitoneal dissemination and distant metastases [8].

Contemporary diagnostic standards categorize typical leiomyomas as masses displaying homogeneous low signal on both T2WI and DWI. These usual or ordinary leiomyomas can be classified as Type 1 in the Funaki classification [9]. Nonetheless, as many as 65% of leiomyomas exhibit degenerative alterations [10], leading to a deviation from this characteristic MRI appearance. Consequently, distinguishing them from uterine sarcomas can pose challenges, with histopathological findings revealing that between 0.01% and 0.5% of tumors initially diagnosed as leiomyomas are ultimately confirmed as sarcoma postresection [11, 12].

Our two reports emphasize that such uterine masses should not be classified as “certainly benign” solely based on the presence of a global or focal area of low signal on T2WI equal to that of pelvic muscles or gluteal muscles. In particular, the mere presence of a focal area of low T2 signal within a heterogeneous mass appears to be considered a weak indicator in a recent consensus article [13]. This classification should not be made without ensuring that there are no intratumoral areas with a high b1000 DWI signal intensity increasing compared with b0-weighted sequence, as overlooking this possibility may result in missing a diagnosis of sarcoma, leading to a worsening of the prognosis for the patient. Additionally, this recommendation extends to cases where the tumor presents areas with a low T2 signal due to hemorrhagic necrosis (a.k.a. “T2 dark” areas), which can be identified by its corresponding high signal on T1WI with Fat-Sat and lack of enhancement after injection of contrast medium [14]. In the radiological literature, rare cases of uterine sarcomas with a solid portion showing low T2 signal have been reported [6, 7, 15]. It should also be noted that there have been previous reports of uterine leiomyosarcoma arising within leiomyomas [16, 17]. In the histopathological literature, this malignant transformation is described even in usual leiomyomas [18–21]. In such rare cases, part of the tumor may show low intensity on T2WI despite being a uterine sarcoma. It has even been reported that a STUMP can present the appearance of a typical leiomyoma with homogeneous low T2 signal (Funaki Type 1), although this occurrence remains rare [22]. In any situation, as in our first case, DWI allows detection of the small malignant focus hidden within the largely benign and/or fibrous component, which could be missed on T2WI. This property is analogous to a “scintigraphic effect.”

In our previous publication [5], we identified three predictive MRI criteria for malignancy in typical uterine sarcomas, with these criteria applying only to solid enhancing regions of the mass:

- First, an intermediate or high signal on T2WI, either homogeneous or heterogeneous. An intermediate T2 signal was defined as a signal between pelvic muscles and adipose tissue, while a high T2 signal was considered similar to that of adipose tissue or bladder urine. These signal characteristics on T2WI in smooth muscle tumors of the uterus resemble those of Types 2 and 3 of the Funaki classification [9].
- Second, a high signal on DWI at high b values, increasing from b0- to b1000-weighted sequence. A high b1000 DWI signal was defined as a signal higher than that of the outer myometrium (i.e., the plexiform layer).
- Third, an ADC restriction of less than or equal to 0.86 × 10^−3^ mm^2^/s (sensitivity = 73%, specificity = 92%, positive predictive value = 92%, overall accuracy = 81%). In the case of a region with high b1000 DWI signal, we recorded the ADC value by placing a circular region of interest (ROI) (default size: 12 mm^2^) on the tumor's ADC map in the area with the lowest mean ADC value, coinciding with the subregion showing the highest b1000 DWI hyperintensity, while avoiding hemorrhagic, necrotic, or cystic areas within the lesion by referring to T1WI with Fat-Sat, T2WI, and contrast-enhanced images.

In doing so, we observed some limitations in the utilization of each MRI sequence. Some leiomyomas may exhibit solid portions with restricted diffusion. Some sarcomas may demonstrate solid portions with low signal on T2WI, as in our two cases, or low signal on b1000 DWI; however, a given sarcoma is never hypointense on both of these sequences simultaneously. Additionally, in extensively hemorrhagic masses, solid portions of a sarcoma may demonstrate relatively lower b1000 DWI signal intensity. This occurs when the b1000 DWI signal from the hemorrhagic portion is extremely high, which can overwhelm the overall DWI signal intensity, as the gray levels are calibrated to the signals present in the image. On the other hand, extensive intratumoral necrosis may cause a leiomyosarcoma to exhibit low signal on b1000 DWI [15].

Ultimately, these results illustrate the diagnostic benefit of systematically combining analysis of tumor signal on T2WI, T1WI with Fat-Sat, DWI, and quantitative measurement of ADC. Thus, it might be possible to devise an MRI scoring system utilizing the identified significant features, as has already been done for other mesenchymal tumors [23, 24].

Of course, morphological features on nonenhanced and postcontrast MRI sequences must also be taken into account: Invasive tumor contours, enlarged lymph nodes (i.e., small axis of 10 mm or greater), or peritoneal implants suggest malignancy.

Exercise caution when interpreting DWI, especially in cases of uterine myxoid leiomyosarcoma, a rare yet distinctive malignant tumor. Myxoid tissue, characterized by its signal resembling that of fluids on T1WI and T2WI, notably exhibits pronounced T2 hypersignal on MRI. Contrast-enhanced imaging reveals signal enhancement due to the vascularized nature of the tumor tissue, distinguishing it from cystic lesions. Histopathologically, tumors are classified as myxoid if they contain an abundant myxoid extracellular matrix occupying at least 50% of the tumor area. Histomorphological features typical of conventional leiomyosarcoma may be subtle in the myxoid variant, particularly with extensive myxoid matrix [25]. Our experience suggests that myxoid leiomyosarcomas may lack restricted diffusion on MRI. Indeed, the mean ADC value has limited ability to discriminate between benign and malignant myxoid-containing tumors, as it can be influenced by the extracellular matrix containing a large amount of water, as well as by tissue cellularity [26]. However, the presence of an infiltrative tumor border remains a consistent diagnostic criterion for malignancy and should be routinely assessed.

Eventually, in the context of smooth muscle tumors of the uterus, MRI is part of a probabilistic approach, and no radiodiagnostic algorithm is absolutely accurate. The radiologist's role is to highlight the possibility of a nonbenign tumor to avoid inadvertent tumor morcellation. Although there are some exceptions, benignity can be considered certain in the case of a solid tumor displaying homogeneous low signal on T2WI and b1000 DWI simultaneously, or intermediate signal on T2WI and no hyperintensity on b1000 DWI. In all other scenarios, caution is advised. Recent advancements in oncogenomics, coupled with the development of coaxial needle percutaneous biopsies, will allow for much greater diagnostic reliability in atypical or suspicious myometrial masses on MRI in the future [27].

## 4. Conclusion

In light of our previous publication [5] and the insights gained from these two clinical cases, it appears that in MRI characterization of a uterine smooth muscle tumor, particularly when examining a leiomyoma variant, especially if heterogeneous, DWI emerges as the “dominant” sequence, while T2WI serves as the “secondary” sequence, to borrow a terminology akin to the PI-RADS. When encountering an intratumoral area exhibiting a high b1000 DWI signal, assessing the lowest mean ADC value enables us to propose a histopathological hypothesis based on the following scheme—a.k.a. “the Hôtel-Dieu (HTD) scheme”—: leiomyosarcoma ≤ ADC = 0.86 × 10^−3^ mm^2^/s > STUMP, cellular leiomyoma ≤ ADC = 1.23 × 10^−3^ mm^2^/s > cellular leiomyoma, edematous leiomyoma [5]. However, it is important to keep in mind that while a significant difference in ADC values between sarcomas and leiomyomas is described, there remains a partial overlap with STUMP, cellular leiomyomas, and edematous leiomyomas in the ADC range between 0.86 × 10^−3^ mm^2^/s and 1.23 × 10^−3^ mm^2^/s; a particular caution is required for an ADC value ≤ 1.10 × 10^−3^ mm^2^/s [15, 28]. In this setting, ancillary imaging features may provide additional information, which can be used for further lesional characterization, and hence increase diagnostic accuracy [29]. Thus, radiologists should be aware of the probabilistic approach inherent in the HTD scheme and remain vigilant, as “low-grade leiomyosarcoma” could be considered if the ADC is ≤0.905 × 10^−3^ mm^2^/s but >0.86 × 10^−3^ mm^2^/s [4, 30, 31].

## Data Availability

The data used to support the findings of this study are available from the corresponding author upon request in order to protect patient privacy.

## Nomenclature

ADC: apparent diffusion coefficient
DWI: diffusion-weighted imaging
ER: estrogen receptor
Fat-Sat: fat saturation
FISH: fluorescence in situ hybridization
HTD: Hôtel-Dieu
MRI: magnetic resonance imaging
PI-RADS: Prostate Imaging Reporting and Data System
PR: progesterone receptor
ROI: region of interest
STUMP: smooth muscle tumors of uncertain malignant potential
T1WI: T1-weighted imaging
T2WI: T2-weighted imaging
